# Older persons’ lived experiences of being playful in nursing home settings – a phenomenological reflective lifeworld research study

**DOI:** 10.1080/17482631.2026.2645255

**Published:** 2026-03-14

**Authors:** Anna Bergman, Maria Haak, Karin Örmon, Anna Nivestam, Albert Westergren, Petra Nilsson Lindström

**Affiliations:** a Faculty of Health Sciences, Kristianstad University, Kristianstad, Sweden; b Department of Health, Blekinge Institute of Technology, Karlskrona, Sweden; c The Västra Götaland Region’s competence centre on intimate partner violence, Gothenburg, Sweden; d Swedish Red Cross University, Huddinge, Sweden

**Keywords:** Ageing, caring, caring science, health science, nursing homes, older adults, phenomenology, play, reflective lifeworld research, well-being

## Abstract

**Background:**

Being playful and having the capability to play are considered fundamental aspects of being human and are closely linked to well-being in adulthood. Despite the health-promoting potential, being playful has, to our knowledge, not been explored in Scandinavian contexts in relation to older persons with functional impairments, such as those living in nursing homes.

**Objective:**

This study, therefore, aims to explore older persons’ lived experiences of being playful in nursing homes, to gain an in-depth understanding of the phenomenon and to contribute knowledge that may support person-centred care and well-being.

**Methods:**

This phenomenological study is grounded in a reflective lifeworld research approach. Lifeworld interviews were conducted with 15 older persons aged 68–100 years.

**Results:**

The essential meaning of the phenomenon emerges as *getting in touch with an inner dynamic life force that enables and enhances well-being*. This meaning is further illuminated through four constituents: engaging in timeless inner wanderings, adapting to bodily change, opening towards belonging, and navigating in a state of dependency.

**Conclusions:**

In older persons´ playful mode of being, the inner dynamic life force opens up to a profound sense of existential well-being. However, ageing, bodily changes, and institutional constraints shape how playfulness is expressed and manifested in the lifeworld, thereby influencing well-being and human dignity. Taken together, these findings point to the potential value of acknowledging playfulness in future research and care practice as a fundamental aspect of being human and a contributor to both well-being and human dignity.

## Introduction

Adult playfulness has been suggested to be strongly related to positive emotions, predicting subjective well-being (Farley et al., 2021; Lubbers et al., 2023) and to an interest in engaging in various activities (Lubbers et al., 2023). It also appears to be important in managing stress (Clifford et al., 2024; Magnuson & Barnett, 2013), and playful older persons have reported higher levels of happiness (Proyer, 2014). Furthermore, being playful in social situations is associated with feelings of being loved and supported by others. To illustrate, older persons who express their playfulness socially are more likely to experience overall well-being, including positive emotions, and greater relationship satisfaction (Brauer et al., 2024; Parker et al., 2023). Additionally, engaging in playful activities is described by older persons as a way to maintain good health, stay active, and keep the mind sharp (Burr et al., 2019), and it has been proposed to emphasise playfulness as a means of enhancing the health and well-being of older persons (Waldman-Levi et al., 2018). Despite evidence highlighting the health benefits of playfulness, research on playfulness among older persons within caring science remains limited, indicating a need for further exploration of this phenomenon.

Given the potential link between playfulness and well-being among older persons, well-being is understood here as a subjective experience of feeling well, regardless of the presence of disease (Eriksson, 2018). In addition, well-being is conceptualised as a holistic state of health, manifested as an overall experiential state of being in balance with others and with life as a whole (Dahlberg & Segesten, 2010). From this perspective, playfulness emerges as a potentially meaningful dimension of health and well-being in later life. As such, playfulness may be regarded as an important area of enquiry, particularly in light of the World Health Organisation’s (WHO) call to strengthen and transform how countries provide care and social support for older persons (WHO, 2024). Central to this transformation is the provision of person-centred and integrated care that responds to the needs of this target group. In Sweden, the core values guiding the care of older persons emphasise the right to live with dignity and to experience well-being (National Board of Health and Welfare, 2012). These values are grounded in an ethical vision of care and social support that extends beyond medical treatment and aligns with the ethical foundation of person-centred care. As described by Ekman, (2022), drawing on Ricoeur (1992, p. 172), this foundation is articulated as *“aiming at the ‘good life’ with and for others, in just institutions”.*

Despite these ambitions, a recent self-rated health survey for older persons receiving care and social support in Sweden revealed that only 32% estimated their health as good (National Board of Health and Welfare, 2024). These findings underscore the need to develop approaches that can enhance well-being within care and social support. Although playfulness has been associated with health-promoting effects, research exploring this phenomenon within caring science remains limited, particularly in nursing home settings. In this context, nursing homes primarily aim to support the everyday lives of persons who are, to varying degrees, functionally impaired, dependent, and vulnerable (Quehenberger & Krajic, 2017).

Thus, questions remain concerning the meaning of being playful in the everyday lives of older persons living in nursing home settings. Meaningful situations constitute a central concern in both person-centred care (McCormack & McCance, 2017) and caring science (Dahlberg & Segesten, 2010). Through lifeworld theory, person-centred care can deepen our understanding of what it means to be human, particularly in vulnerability (Galvin & Todres, 2013). Although factual limitations shape our lives, they do not ultimately define who we are. We are always self-making beings, through the meanings we give to our choices (Aho, 2021). Within caring science informed by a lifeworld perspective, this continuous tension between determinism and freedom is understood as an interplay between vulnerability and freedom (Dahlberg & Segesten, 2010; Galvin & Todres, 2013).

Previous research on playfulness and well-being has primarily focused on adults and older persons without functional impairments or care dependency. However, from a lifeworld perspective, functional impairment reframes the experience of the lived body and gives rise to a different way of being in the world, a changed relationship to time, space, the environment, and others (Merleau-Ponty, 1945/2002). Hence, being playful as an older person in nursing homes cannot be understood only as an individual trait or behaviour, but as something that emerges in the interplay between the person’s vulnerability and freedom, body and environment, and the meanings that arise in everyday life. This raises important questions about how such meaningful ways of being playful are experienced.

## Aim

The present study aimed to describe the lived experiences of being playful among older persons living in nursing homes.

## Method

This study is part of the larger research programme *Play and Supportive Environments* (PLAY-SE), which aims to define the interrelated concepts of being playful and playful activities among older persons receiving care and social support, for example in nursing home settings (Westergren et al., 2025).

### Design

This phenomenological study is based on a reflective lifeworld research (RLR) approach described by Dahlberg et al. (2008). RLR is grounded in phenomenology and hermeneutic philosophy, drawing on Husserl’s theory of the lifeworld and intentionality (Husserl, 1936/1970), Merleau-Ponty (1945/2002; 1964/1968) theory of the lived body and the flesh, as well as the hermeneutics of Gadamer, 1960/2004).

RLR is meaning-oriented, and the philosophical underpinnings provide methodological principles such as openness, bridling, and flexibility (Dahlberg et al., 2008). Thus, the researcher needs to remain open and flexible. The bridled attitude involves continuous self-reflection on personal beliefs and theories, given that these may influence the understanding of meaning and thereby hinder the phenomenon from fully revealing itself (Dahlberg et al., 2008). By adopting these principles throughout the research process, the researchers returned to the things themselves, that is, to the phenomenon under study: *being playful when living in nursing homes as experienced by older persons.*

### Setting

In accordance with the Social Services Act (SFS 2001:453), the municipalities are responsible for providing care and services in Swedish nursing homes. Assistance with personal care is one of the most common supports provided in Swedish nursing homes (National Board of Health and Welfare, 2023). Additionally, the care and support provided often lie at the intersection of social services and healthcare (Quehenberger & Krajic, 2017). Personnel employed in nursing homes in Sweden comprise care assistants, assistant nurses, registered nurses, occupational therapists, and physiotherapists.

The present study was carried out in two municipalities in southern Sweden, encompassing six nursing homes, of which one was privately operated. The nursing homes were located in both rural and urban areas. The facilities varied in size, ranging from smaller to larger units. None of them was exclusively somatic; all included units for persons with cognitive impairment. In some wards, residents with cognitive impairment and residents with somatic illnesses lived together. The activities arranged at the facilities were intended solely for the residents, and some included residents from both dementia and somatic units. Some facilities had dedicated activity coordinators on site, whereas others either lacked such staff altogether or only had them present periodically.

### Recruitment process and participants

A purposive sample was adopted to achieve diversity of persons residing in both rural and urban areas. Inclusion criteria were persons 65 years and older, living in nursing homes and capable of providing informed consent. Participants were recruited following the first author’s communication of information, via e-mail, to the managers at the nursing homes. Subsequently, in all but one nursing home, the managers were requested to convey information to personnel who served as gatekeepers, and the persons residing in nursing homes received oral information about the study from the personnel. At one nursing home, oral information was presented by the first author. For those interested in being part of the study, written information was provided together with a consent form. The time and location of the interviews were arranged together with the older persons, either directly by the first author or through the personnel acting as intermediaries. All participants chose the location they found most suitable for the interview, and in every case, this was their own apartment within the nursing home. Participants were informed that they could withdraw from the study at any time without providing further explanation.

A total of 18 persons declared an interest in being part of the study. Two persons later withdrew their interest without further explanation, and one withdrew due to acute illness. Ultimately, 15 persons, nine women and six men, were interviewed about their lived experiences of being playful. Their ages ranged from 68 to 100 years old, with a mean of 89 years. Their lived experiences of being playful while living in nursing homes varied between three months and eight years, with a median of one and a half years.

### Procedure and data collection

A reference group was established prior to data collection, encompassing two older persons residing in nursing homes. The reference group collaborated with the first author in the study, for example, through the formulation of the initial research question on the phenomenon.

The lifeworld interviews were conducted by the first author between February and April 2025. All interviews were conducted face-to-face in the participant’s apartment at the nursing home. To direct the participant’s attention to their everyday lived experiences, each interview was initiated with an opening-up question about their daily life in the nursing home setting, in line with the RLR interview approach (Dahlberg et al., 2008): “Tell me briefly about a day in your life at the long-term care facility”. This was followed by a question about the phenomenon under study: “Tell me about how you are being playful in your everyday life”. When necessary, the opening-up question on playfulness was supplemented with the question “What do you do for amusement?” The interviewer followed the person being interviewed, and descriptions and narratives were supported when the phenomenon was in focus. This support took the form of follow-up questions to move towards the unknown and unconsidered about being playful, such as: “What do you mean by…”, “In what way…”, “How was it good?”, “What does that mean to you?” The interviews lasted between 33 and 87 minutes (mean = 59 minutes). All interviews were audio-recorded and subsequently listened to and transcribed verbatim by the first author.

### Data analysis

The transcripts were analysed for meaning in accordance with the RLR principles of openness, flexibility, and bridling (Dahlberg et al., 2008). The transcribed text was read several times by the first and last author to gain an overall sense of its meaning. At this stage, openness was essential, as was maintaining a receptive stance towards the text and what it might convey. The task was to interrogate the text to disclose new insights and view the material from a different perspective, allowing the text to “speak” to the researchers. This process required a sense of immediacy, thus being close to the text while also remaining curious and open to surprises. When the text was familiar as a whole, the reading changed, and meaning units—that is, the meanings related to the phenomenon—were noted. Meanings were compared based on differences and similarities and subsequently sorted into patterns of clusters. The analysis proceeded with a search for underlying patterns of meaning, and the individual meanings were compared with clusters in the “figure and background” approach, in the in-between space, between the researcher and the phenomenon, (Dahlberg et al., 2008). A bridled attitude was adopted in seeking the essential structure of meaning—the essence. The essence “is” the phenomenon, the phenomenon’s style and way of being. The constituents are particulars of the essence, that is, the phenomenon’s inner and outer horizons; therefore, the essence must be seen in every constituent (Dahlberg, 2006).

The first and last author searched for meanings in the interview transcripts. These meanings were first considered individually and then discussed together. Individual analyses were followed by joint meetings where the meanings and clusters were discussed with a phenomenological attitude and an awareness of pre-understanding. Finally, the authors discussed the findings together. The authors are researchers in the field of health science with a variety of occupational titles. The first author (A.B.) is a specialist nurse in the care of older persons and has experience of working in nursing homes. The last author (P.N.L.) is a public health educator. Two authors are registered nurses (A.W. and A.N), one a specialised psychiatric nurse (K.Ö.), and one a registered occupational therapist (M.H.).

### Ethical considerations

The study received ethical approval from the Swedish Ethical Review Authority (Reg. No. 2025-00211-01) before commencement. All participants provided informed consent before participation. At the beginning of each interview, participants were reminded of the study information and reassured that they could withdraw at any time without providing a reason. Ethical considerations were made throughout the research process, in accordance with the Swedish Ethical Review Act (SFS 2003:460) and the Declaration of Helsinki (World Medical Association, 2025).

A potential risk identified was that the research questions might evoke distressing memories or difficult emotions related to engaging in playfulness. To minimise this risk, the researcher offered follow-up contact with the participants a few days after the interviews to ensure that they were not left without support in processing any challenging emotions. Another potential risk identified was that discussing playfulness could be perceived as childish or even provocative. This was discussed with the reference group, and it was jointly concluded that the likelihood of such a risk was minimal.

## Results

The essential meaning of being playful, as described by older persons living in nursing homes, emerges as getting in touch with an inner dynamic life force that enables and enhances well-being.

This inner dynamic life force is understood as a natural, intrinsic energy that is always present within. However, the expression of playfulness transforms during the lifespan and takes on different forms due to both bodily changes in older life and contextual factors of living in nursing homes. How older persons are playful is highly individual, and each person has their own way of playful expression. Consequently, being playful is understood as a mode of being, that manifests as a state of mind and is expressed in different ways in relation to the lifeworld, through memories and in activities.

What unites the different manifestations is the pursuit of amusement. However, although the surface-level purpose is about having fun and amusing oneself, the well-being that arises from being playful transcends the feeling of merely having fun. Being playful is essential for escaping into a parallel imaginary world of enjoyment and temporarily leaving behind the everyday ordinary life. It thus becomes a source of profound well-being, eliciting emotions that transcend mere amusement and foster a sense of capability and belonging. At the same time, being playful functions as a form of protection, helping to safeguard one’s integrity in situations where dependency might otherwise undermine it. Since the meaning of being playful is closely connected to the experience of well-being, the older persons strive to be playful. This striving reflects expressions of playfulness that are continuously reshaped by the dependency structures that characterise life within nursing home settings. The dependency associated with living in nursing homes means being playful through playful activities that are not always self-chosen; however, the activities can still be experienced as playful in the moment and thereby contribute to an enhanced sense of well-being. The essential meaning of the phenomenon under study is further described by the following four constituents: **Engaging in timeless inner wanderings**, **Adapting to bodily change**, **Opening towards belonging**, and **Navigating in a state of dependency.**

### Engaging in timeless inner wanderings

Engaging in timeless inner wanderings implies an inner continuum of being playful. This inner way of being playful arises from the capacity to remember and relive playful memories from earlier in life. It provides a continuity across time, which connects the past and the present. It is a form of self-directed inner way of being playful, engaging with memories from childhood, adolescence, and middle adulthood. These memories can be summoned in moments of solitude. When memories come alive, the past becomes present and nurtures well-being.

*Well, it’s when you just sit and think, mostly in the evenings. … Oh, what fun we had when we were there. … Yeah, it feels good inside, it really does.* (9)

Playful memories are also re-experienced in conversations with others, in the storytelling and sharing of such memories, where being playful through remembrance brings a sense of embodied well-being in the moment.

*Well, we talk about what it was like before, you know, what we did when we ran around in the monastery … and played hide-and-seek. … Well, we think … it makes you feel all warm inside.* (1)

Timeless inner wanderings are also facilitated in arranged weekly or monthly activities within nursing homes. These activities may be organised by staff, sometimes in collaboration with other residents, but also by voluntary organisations or relatives of fellow residents. Regardless of who arranges them, a way of being inwardly playful emerges when accompanied by feelings that connect to playful memories from earlier stages of life. Thus, what relates to such playful memories varies from person to person. The memories can be sparked by familiar songs, narrative stories in books, or movements that bring the past vividly into the present and nurture a profound sense of enjoyment, as illustrated in the following quote about the feeling of rhythm in the body when listening to music:

*Yes, of course, you sit there clapping, and when something is really good and rhythmic you keep doing this [taps their knees rhythmically], and then there’s a little [pause], then memories and such come flooding back, and they really do dance in! … everything becomes lighter. It can’t be helped. I was young, and I was mobile, and I almost said, everything was fine!* (13)

Engaging in timeless inner wanderings also suggests that being playful unfolds across a future timespan. In this sense, a playful activity extends beyond the immediate moment of engagement, offering a duration of being playful. It encompasses activities extending over longer timeframes, such as seasonal activities. Although a playful activity may be physically enacted in spring or summer, it begins much earlier, when it is imagined and carefully planned during the winter months. Anticipation and preparation nurture feelings of joy, and being playful begins inwardly long before it becomes outwardly visible, fostering a sustained sense of well-being.

*… there we’re going to build a trellis so that we can tie the tomatoes to it … have sticks that go nice and high, you know. I’m going to start doing things like that soon, so I think about such things, and I almost find that—doing that—to be playful. For me, it’s playful. … Well, it’s something I’m really interested in, you know, that’s where I feel so damn good, getting my hands in the soil, you see.* (2)

### Adapting to bodily change

Adapting to bodily change implies a gradual adaptation of expressions of playfulness in relation to the body’s functions. This is particularly evident in cases where the body has served as the primary vehicle for being playful. It is about adapting the body to one’s project of being playful. However, it is sometimes not possible to continue with the project due to bodily changes, and other playful expressions that resonate with the body are chosen.

When the adaptation is in progress, the body restricts the possibilities for playful expression by limiting the likelihood of being playful spontaneously, thereby narrowing the playful world. Over time, however, a habituation takes place, in which the body is no longer experienced as an obstacle to the same extent as before. This is not due to the return of physical ability, but rather to becoming accustomed to being playful in new ways, in ways that resonate with the body. Yet, the playful world is nevertheless diminished by bodily change, inhibiting the spontaneous expression of playfulness, as described below:

*Yeah, now I’ve been this disabled for so long that I’ve sort of adapted to it. I think about myself as I am. In the beginning it was hard.* *“Oh, I’ll fetch that in a jiffy” but oh no, that’s it, I can’t walk. Then the sphere shrinks, like a dark hole, a black hole. Like that [claps their hands together]. There’s nothing left.* (3)

Simultaneously, the bodily expressions of playfulness are reshaped to make playful activities possible. Being given the opportunity to adjust one’s bodily expressions to activities that one used to enjoy earlier in life, during youth and middle age, can evoke embodied feelings of joy. As a result, being able to continue with one’s playful project in individualised activities may foster feelings of happiness and amusement, as illustrated in the following quote:

*Yeah, the thing is, you’re older, you’re older. You can’t have real gymnastics when you can’t move, you know, instead you must sit on a chair and do some tricks, and then we throw a ball. And that’s something … I’ve always really, really loved throwing a ball. … It’s in the body. … It´s a nice feeling. … Some kind of, how should I say—joy in the body [laughter]!* (9)

When adapting to bodily changes, assistive devices serve as enablers of playful expressions, supporting the continuation of being playful. Yet, their use also calls for a process of habituation and adaptation, through which the body gradually learns to attune to the devices. In this process, the very manner in which playfulness is expressed through the body is slowly reshaped, opening new but altered pathways for bodily expressions. Such gradual adjustments are, for some, vital for continuing with one’s playful project from earlier in life—before bodily changes—and may foster feelings of capability and a sense of mastery.

*I also play boules. Boules—you know what that is, don’t you? Others throw like that [makes a throwing motion], but I must somehow lean outside of my wheelchair and throw like this. I lost my legs. … So, I’ve got no choice. I just had to learn something new, how to go about it. I lean outwards like this and arrange for the wheel not to be in my way, so I’ve become pretty good at it too!* (5)

Adapting to bodily change also implies that it is sometimes necessary to focus on other playful activities, such as those more suitable to the body. When the body is unable to carry out an activity independently, for example, due to pain, the activity can no longer be performed, and alternative playful expressions, less reliant on the body, are adopted, even as a longing for one’s original playful activity remains.

*It hurts like hell! … Yeah, I hope that, in some darned way, I want to get there again.* (13)

### Opening towards belonging

Opening towards belonging implies being playful in relation to others, presupposing mutuality in playful expressions. Thus, a reciprocal expression of playfulness is essential for playfulness to hold meaning in relation to others. It is grounded in relationships with significant others in life, such as fellow residents, staff members, one’s own relatives, the relatives of other residents who engage in playful activities during visits, as well as volunteers who are welcomed into the community of the nursing homes. Expressions of playfulness need, however, to be in harmony with those of the other person; only under such conditions can mutual playfulness arise. Such harmony does not occur with everyone, yet only through it can playfulness be genuinely experienced with significant others, giving rise to a playful bond and a sense of belonging.

*There is one carer. He works nights. We usually have a laugh together. But he hasn’t been here in four–five days. … I miss him. … then he stands there in the doorway, and it makes me happy when I see him, I think: ‘Now I can have a joke with him.’ …* (6)

The belonging formed through playfulness may be momentary, but can also develop into a close, long-term friendship. When seeking playful connectedness with others, sensitivity is required to recognise with whom playfulness is possible. Not all interactions allow for playfulness, and sometimes there is a risk involved when playfulness is directed towards the wrong person, for example, through a joke that is misunderstood. Nevertheless, taking such a risk can be worthwhile, as successful playfulness promotes mutual positive experiences and creates a sense of well-being and belonging, which extends beyond the spontaneous speech of the other.

*Only the other day she said it again. ‘You don’t know what it meant to me when you arrived, you arrived like a ray of sun.’ … She’s still deadly quiet, so she’s sort of unable to start a conversation by herself, but when I fool around, oh boy, then she laughs so heartily!* (4)

The potential for mutual playfulness and a sense of belonging is, however, constrained when living alongside persons with severe cognitive impairment. Direct playful interaction with these persons is not possible, as they inhabit a different playful world. Moreover, the considerable time and attention required from staff members by persons living with severe cognitive impairment leads to an absence of spontaneous playfulness that might otherwise unfold in everyday interactions between residents and between residents and staff. Within this context, the role of activity coordinators becomes vital, as they provide a dedicated space where playfulness can be nurtured and experienced as a form of belonging.

*And then there’s this old bloke sitting there shouting ’Mum, mum, mum’! Such patients shouldn’t be here. There should be people that you could have a bit of a chat with and who can, maybe they can’t walk or whatever, or they’re in a wheelchair, but clear-headed enough that you could play games together and that kind of thing. But there’s not much of that, but, well, Sofie [the activities coordinator] does come. … and then she’ll bring some games and quizzes, and stuff like that. Yes, that’s nice! … Being together with other people!* (8)

### Navigating in a state of dependency

Navigating in a state of dependency involves being playful in a continuous balancing act between internal and external demands. Playfulness may function as a protective resource that safeguards one’s inner value against internal demands, yet it is constrained by external circumstances such as institutional guidelines, routines, and legal requirements.

When navigating in a state of dependency, engaging in a challenging game that requires concentration and problem-solving constitutes an essential task in protecting internal demands. In being able to carry out such playful activities, a sense of acknowledgement of one’s value is generated, reinforcing feelings of being capable.

*Then you must think a bit more, not just sit around here, reading and watching the telly, but think for yourself a little, with quizzes and stuff. I feel that my brain still works quite well and that I’m still keeping up. [Interviewer: What does that mean for you?] It means that I can.* (8)

Being playful also serves a vital function in protecting personal integrity in moments of intimate care. In such situations, being playful is a means of protection, where playful expressions help preserve bodily integrity, acting as a shield against the vulnerability of exposure in care.

*I thought it was awful when I arrived here, having to show my backside to everyone. You can imagine. [Then the interviewee joked by saying:] Are you going to wash the ‘musikant’ [“musician”; Swedish slang meaning “butt”] now? … I think joking helps in all situations. … Yeah, there’s something in the body that [pause] that is created,… that has a healing effect on the human being.* (15)

Caring for the ageing body is regarded as burdensome, and playfulness in bodily care situations serves not only to protect the older person but also to support staff in the demanding task of providing such care. In this sense, being playful fulfils a twofold function when safeguarding the older person while simultaneously contributing to supporting the staff in their demanding work situation.

*I feel that when I notice that they’re happy and maybe can stand their job better, that gives me a real sense of satisfaction. I feel that I’ve maybe contributed to making them feel better at their job. … It makes up for the feeling of discomfort they’ve had when they’ve been in here turning over a sick person who’s maybe a little demented and such. … It’s good if everyone’s playful and friendly and nice back to the staff.* (7)

Navigating in a state of dependency also entails pressure to conform to external structures, including institutional constraints such as guidelines, routines, and legal framework, conditions that shape how playfulness can be expressed. The extent to which playfulness is facilitated varies across nursing home settings. In settings where playfulness is encouraged, opportunities for personal expression and everyday playful activities are supported, whereas in other settings, such opportunities are limited, resulting in an absence of playful expressions.

*No, that, the playfulness didn’t come till I moved into the home. … This is where I’ve become playful! … I didn’t have the right tools for it.* (2)

Even in nursing homes where playfulness is explicitly encouraged, a state of dependency gives rise to situations in which playfulness cannot be fully expressed. This is because supporting older persons in engaging in self-chosen playful activities that they are unable to perform independently lies beyond the established routines and designated responsibilities of staff. Thus, opportunities for being playful rely on the involvement of people from outside the facility. In such situations, assistance from a significant person outside the facility becomes particularly important for maintaining opportunities for playful activities—something that is usually understood and accepted by the older persons, although longing for the playful activity remains. Being playful is thus constrained by institutional culture, affecting the older persons’ possibilities of being playful.

*If we hadn’t been here—us disabled and old people—this would’ve been a great place! There’s bloody EVERYTHING here! … No, but we [pause] we don’t really use it, do we? We aren’t able to. … The way they’re running around, you just can’t pile anything else on them. No, no, I’ve worked myself, so I know what it’s like when, when you´re short on time. [Interviewer: What does it feel like that they don’t have time to help you express your playfulness?] Well, it’s a bit rough, of course [pause] But, erm, I realise, you know, just like when I was working, that it’s impossible.* (13)

When navigating in a state of dependency, arranged playful activities that are not self-chosen can serve a vital function. Life in nursing homes often consists largely of waiting for assistance in activities of daily living, such as toileting, eating, and other basic needs. It also consists of waiting for dying, with feelings of isolation further reinforcing this sense of waiting, as daily life for some is characterised by restricted freedom of movement both indoors and outdoors. In some nursing homes, being playful outdoors is not possible. Days when playful activities are arranged offer a temporary pause from everyday life.

*Yes but, we’re here, aren’t we? … Everyone must know where I am. … But, er, be that as it may, I know where I’m heading. … It [organised playful activities] breaks up the day a bit, the usual one. You know, we do live a pretty dull life, really. You know, our lives are actually quite dreary. Yeah, there’s no getting away from that. We are after all in an institution. … Yes! We’re in this kind of, well, we’re not in a prison, exactly, but we’re not far off. Do you see what I mean?* (1)

## Discussion

The present study aimed to describe the lived experiences of being playful among older persons living in nursing homes. To our knowledge, this is the first study to provide an in-depth understanding of being playful as experienced by older persons living in nursing homes, thereby contributing new insights into this phenomenon. The older person’s way of being playful in the lifeworld, in the tension of freedom and vulnerability, emerges as getting in touch with an inner dynamic life force that enables and enhances well-being. From an existential and lifeworld perspective, this life force is understood as vitality, an existential form of well-being characterised by possibilities of *movement* and possibilities of *peace* (Dahlberg & Segesten, 2010; Galvin & Todres, 2013).

The findings indicate that possibilities of existential *movement* arise when being playful. In playful moments, the older person moves between different times and places through timeless inner journeys. Also, a sense of belonging, agency, and capability emerges that protects human dignity in dependency and vulnerability. From an existential and lifeworld perspective, this is understood as the capacity of *movement* in the sense of being able to move into possibilities of engagement that connect us with others, other spaces, other times, and other moods (Galvin & Todres, 2013). Also, when escaping into a parallel playful world, everyday constraints are forgotten, if only for a fleeting moment, in the playful act. Thus, in the movement, a sense of *peace* emerges, as everyday constraints are momentarily suspended and a “letting be” unfolds. This aligns with Gadamer’s view that, in play, the person finds rest from the instrumental rationality that structures everyday life (Gadamer, 1960/2004). Thus, the findings demonstrate that having the opportunity to be playful serves a vital function in experiencing a profound, intertwined existential well-being of movement and peace in nursing home settings. An overall sense of experiential health is achieved by being in balance with others and with life as a whole (Dahlberg & Segesten, 2010), if only in the fleeting moment while being playful.

Furthermore, the findings show that engaging in timeless inner wanderings by recalling playful memories from earlier in life plays a vital role in being playful in later life. Although this life-affirming force of memory can be accessed in solitude, the findings also indicate that sharing one’s playful life story with another person can evoke playful memories. In addition, during arranged activities in nursing homes, movements or familiar rhythms can spark playful memories, allowing embodied well-being to enter vividly into the present moment. What evokes such memories varies from person to person; however, it points to an important temporal aspect that staff in nursing home settings should be aware of, suggesting that a state of gerotranscendence plays an important role in being playful (Tornstam, 2011). Thus, in gerotranscendence, revisiting playful memories and connecting across generations are key to nurturing playfulness in later life. To promote well-being, it is therefore crucial not to unthinkingly project middle-aged standards onto older persons, but instead to meet their capacities for playfulness. According to Dahlberg & Ekman (2017) and Ekman (2022), one of the greatest forms of suffering occurs when a person’s capabilities go unrecognised in the care context (Dahlberg & Ekman, 2017; Ekman, 2022). Furthermore, having the opportunities to play and be playful is considered strongly linked to human dignity (Nussbaum, 2011). Recognising the older person’s capabilities and listening to their narratives about being playful are therefore important aspects of person-centred care in nursing home settings, as this can promote well-being and human dignity.

Although the playful world is diminished due to bodily changes, the possibility to continue with one’s playful project is important for some in experiencing well-being. Thus, having the opportunity to engage in self-chosen individualised activities, attuned both to the body and to one’s project of being playful, can lead to an embodied sense of well-being. To be able to take part in such individualised self-chosen enjoyable activities in nursing homes has also been reported in earlier studies to be a crucial factor in experiencing overall human dignity (Slettebø et al., 2017). Thus, providing individual and playful activities in nursing homes is considered important to align with the core values in the care of older persons in Sweden, namely, the ability to live with dignity and to experience well-being (The National Board of Health and Welfare, 2012). Traditionally, the assessment of functional abilities in activities of daily living in nursing home settings is addressed using the *Instrumental Activities of Daily Living* (I-ADL) and *Personal Activities of Daily Living* (*P*-ADL) (Hartigan, 2007). The present study’s findings suggest that the older person’s way of being playful should be regarded as a vital, yet often overlooked, component in the assessment of *P*-ADL. While the ability to perform daily activities is typically assessed in functional terms, our findings indicate that playfulness can be understood as part of the person’s lived capacity to engage meaningfully with life. In person-centred care, establishing a partnership between older persons and staff is crucial for developing care plans that genuinely meet individual needs. The first step in this partnership involves eliciting the person’s narrative, understood as their lived experience of illness and its impact on daily life (Ekman, 2022). However, our results indicate that in practising the person-centred ethical intention as ‘*aiming at the “good life” with and for others, in just institutions’* (Ricoeur, 1992, p. 172), the focus of care and solicitude in nursing home settings needs to extend beyond the older person’s perceptions of illness and functional ability. It should also encompass their overall well-being, particularly by recognising and nurturing their need to be playful and play as a life-affirming force that supports dignity and vitality in everyday life.

Furthermore, the results of the present study contain a contradictory finding, related to engaging in non-self-chosen, arranged playful activities within the nursing home. Such a non-self-chosen way of being playful also seems to have the potential of promoting well-being. When the body emerges as an object and restricts one´s possibilities to be playful, the older person cannot rely on the staff to support a self-chosen activity that falls outside the scheduled programme of the nursing home, as it lies beyond the staff’s immediate responsibilities. Here, the freedom in vulnerability becomes evident when the older person finds other ways of being playful in their restricted freedom within the nursing home setting. The limited freedom to be playful is experienced by the older person as part of the ageing body itself, something belonging to its nature. Although the limited freedom to be playful is accepted, a longing for one’s own playful projects endures. This could, of course, be viewed as an acceptance—as a sense of peace, according to the existential theory of well-being (Galvin & Todres, 2013). However, the findings align with earlier research showing that the awareness of staff workload in nursing home settings leads older residents to perceive their influence over daily activities as limited, as they do not wish to disturb the personnel (Jonasson et al., 2023). A contributing factor to this is the institutional culture within nursing homes, which prevents the needs and preferences of older persons from being fully met (Hermansen Østby et al., 2025). Thus, inspired by Martinsen (2006), the findings of the present study need to be viewed with a degree of distance, so as not to risk imposing normative assumptions in ways that may obscure the persons’ lived experiences. To avoid this, we must maintain a certain distance that allows us to truly see the older person as an individual shaped by, but not reducible to, institutional norms. As situated (Beauvoir, 1970/1976) in the flesh of the world (Merleau-Ponty, 1964/1968), this means recognising the older person as embedded in social, historical and cultural frameworks that in silence shape and constrain their ways of being playful in the invisible and mute fabric of meaning in the lifeworld.

The findings also highlight the importance of having significant others to be playful with. Engaging in playful activities with others in the nursing home gives a sense of contributing not only to one’s own well-being, but also to that of the others. Through their agency in promoting well-being when being playful, we see that older persons are active, contributing members of society who foster the well-being of both themselves and significant others, such as staff and fellow residents. Thus, the older persons are important co-creators of person-centred care in nursing homes, all in line with the WHO’s call for actions to strengthen and transform how care and social support for older persons are provided (WHO, 2024). In this sense, it is not about transforming care and social support *for* older persons, but rather *with* older persons. However, being playful with others is constrained when living alongside persons with severe cognitive decline. In Sweden, this is frequently the case, as many residents with severe cognitive impairment live in environments not specifically designed for their needs, due to the lack of specialised nursing homes (Swedish Health & Social Care Inspectorate, 2020). Despite the trend in Sweden for older persons without cognitive impairment to live in ordinary housing, it is still common for them to reside in nursing homes. Thus, living alongside persons with cognitive impairment may narrow the older person’s playful world and restrict the emergence of such fundamental aspects of well-being. Our results corroborate previous research demonstrating positive associations between social playfulness and well-being among older persons (Brauer et al., 2024), and opportunities for playfulness in social situations promote feelings of dignity and a sense of purpose in life (Parker et al., 2023). Thus, these factors are vital for fostering *just institutions*, an ethical cornerstone of person-centred care that calls for fairness and human dignity (Ricoeur, 1992) to be embedded in the design and culture of nursing home environments.

## Methodological considerations

This study is conducted in accordance with the methodological RLR principles of openness, flexibility, and bridling (Dahlberg et al., 2008) throughout the research process. Such an approach seeks to strengthen the evidence of the study while addressing the notions of objectivity, validity, and generalisation (van Wijngaarden et al., 2017). Objectivity in phenomenological research does not exclude the researcher’s involvement: as human beings, we act from within the lifeworld, and thus detached objectivity is never possible (van Wijngaarden et al., 2017). The methodological principle of openness demands that the researcher move beyond the natural, taken-for-granted understanding of meaning in the lifeworld (Dahlberg & Dahlberg, 2019a). This, in turn, requires a bridled reflective attitude, slowing down and resisting the temptation to understand too quickly or carelessly, thereby avoiding the risk of making something definitive that is not (Dahlberg et al., 2008). It requires patience, waiting for the phenomenon to reveal its own complexity. Rather than imposing pre-defined theories, models, or the researchers’ beliefs and wishes, the aim is to let the phenomenon speak for itself (van Wijngaarden et al., 2017). Thus, being aware of one’s own bias when striving to see the otherness (Gadamer, 1960/2004) is essential in this process. A possible limitation of the study concerns the first author’s professional experience in nursing home settings, which may have influenced the evolving understanding of the phenomenon. However, the principles of RLR were adhered to throughout the research process, and the pre-understanding was bridled (Dahlberg et al., 2008), meaning that it was restrained and reflectively examined to safeguard openness towards the phenomenon. In line with this, the researchers maintained ongoing reflective dialogues throughout the research process to grasp the phenomenon and the evolving understanding of it.

The notion of validity in phenomenological research deals with meanings (van Wijngaarden et al., 2017). To illustrate, the questions of the lifeworld interviews were composed so that the persons were directed to focus their attention on their lived experiences of *being playful when living in nursing homes.* Thus, the researcher followed the person, and descriptions and narratives were supported when the phenomenon was in focus. Follow-up questions were used to move towards the unknown and unexamined, that is, beyond the participants' opinions and intentions, to grasp their lived experiences and meanings.

Generalisation in phenomenological research is understood to span the range between the particular and the general (Dahlberg & Dahlberg, 2019b). Thus, presenting the findings through the essence, that is, by first describing what makes the phenomenon a specific phenomenon, and then outlining its particular meanings through its constituents, facilitates the study’s generalisability. Thereby, the findings encompass both the essential, objective meanings and the subjective, particular meanings. In addition, purposive sampling was adopted to gain a wide variety of older persons residing in nursing homes in both rural and urban areas. To facilitate generalisation and determine the applicability in different settings, the context is described in the Method section.

## Conclusion

This study shows that being playful while living in a nursing home is important for well-being. When being playful, a life force awakens feelings of amusement, yet the well-being generated extends beyond mere amusement. It brings a sense of belonging, capability, and agency, and it protects human dignity by offering protection from the constraints of everyday life. Through this life force, older persons can, in their freedom, move towards a sense of intertwined movement and peace that enables and enhances an overall existential well-being.

From a clinical perspective, our findings show that playfulness should not be regarded merely as a pleasant or entertaining activity in later life, but as an existential resource, a life-affirming force that supports human dignity, vitality, and belonging in a life marked by dependency and vulnerability. From a person-centred perspective, this means meeting the older person by listening to their narrative and playful life stories and creating opportunities for being playful in the nursing home. Furthermore, the possibility of being playful in environments not shared with persons with cognitive impairment seems important in supporting well-being. Future research should continue to explore playfulness as a core aspect of existential well-being in nursing home settings—not as an optional addition, but as a fundamental part of what it means to provide high-quality, person-centred care and solicitude in these settings.

## Data Availability

The participants in this study did not provide written consent for their data to be shared publicly. Due to the sensitive nature of the research, supporting data are therefore not available.
